# Lifestyle behaviours in children born extremely preterm at 2 years: a comparison to a Dutch reference population

**DOI:** 10.1007/s00431-026-06986-4

**Published:** 2026-04-29

**Authors:** Nina M. Frerichs, Anna M. le Clercq, Marissa C. J. Kooij, Anne J. A. Krijger, Aranka J. van Wesemael, Rimke R. de Kroon, Chris H. P. van den Akker, Marlou M. A. Raets, Esther J. d’Haens, Willem P. de Boode, Elise Roze, Hans B. van Goudoever, Aleid G. Leemhuis, Berber J. Vlieg-Boerstra, Edgar G. van Mil, Angelika Kindermann, Koen F. M. Joosten, Hendrik J. Niemarkt, Tim G. J. de Meij

**Affiliations:** 1 Department of Pediatric Gastroenterology, Emma Children’s Hospital, Amsterdam UMC, Amsterdam, The Netherlands; 2 Amsterdam Gastroenterology Endocrinology Metabolism Research Institute, Amsterdam, The Netherlands; 3 Amsterdam Reproduction and Development Research Institute, Amsterdam, The Netherlands; 4 Department of Neonatal and Pediatric Intensive Care, Division of Neonatology, Erasmus Medical Center, Rotterdam, the Netherlands; 5 Department of Neonatal and Pediatric Intensive Care, Division of Pediatric Intensive Care, Erasmus Medical Center, Rotterdam, the Netherlands; 6 Department of Pediatrics - Neonatology, Amsterdam UMC, Emma Children’s Hospital, University of Amsterdam, Amsterdam, The Netherlands; 7 Division of Neonatology, Department of Pediatrics, Maastricht University Medical Center+, MosaKids Children’s Hospital, Maastricht, The Netherlands; 8 Department of Neonatology, Isala Klinieken, Zwolle, the Netherlands; 9 Division of Neonatology, Department of Pediatrics, Radboud University Medical Center, Radboud Institute for Health Sciences, Amalia Children’s Hospital, Nijmegen, Netherlands; 10 Department of Pediatrics, OLVG Hospital, Amsterdam, The Netherlands; 11 Department of Pediatrics, Jeroen Bosch Hospital, ‘s-Hertogenbosch, The Netherlands; 12 Maastricht University, Maastricht, The Netherlands; 13 Neonatal Intensive Care Unit, Máxima Medical Center, Veldhoven, The Netherlands; 14 Department of Pediatrics, Maastricht University Medical Center, Maastricht, The Netherlands

**Keywords:** Lifestyle screening, Early childhood, Parental satisfaction, Follow-up, Preterm birth

## Abstract

Children born extremely preterm (EP, < 28 weeks gestation) are at increased risk of lifestyle-related morbidities. This study characterized parent-reported lifestyle behaviours in EP-born children at 2 years’ corrected age (CA) and compared them with a Dutch reference population. In a multicentre prospective cohort study, parents completed the FLY-Kids lifestyle screening questionnaire (2 years’ CA), which assessed parental satisfaction and adherence to age-specific recommendations for diet, eating habits, physical activity, screen time and sleep. Outcomes were compared with a Dutch reference sample of 2-year-olds, and associations were examined using linear regression. Of 269 eligible children, 239 parents responded. After exclusions (*n* = 63), 176 children (74%) were enrolled, of whom 115 (65%) completed the questionnaire (14 twin-pairs). Compared with the Dutch reference population (*n* = 73), lifestyle recommendations were more often unmet in EP children (mean = 3.92 vs. 3.29 items, *p* = 0.01). Interestingly, a larger proportion of EP children exceeded the recommended sleep duration (≥ 14 h) (*p* < 0.001), while other lifestyle items were similar. In both populations, > 30% of children failed to meet the recommendations for seven out of nine items. Higher socioeconomic status (*p* = 0.016) and having at least one foreign-born parent (*p* = 0.034) were associated with lower parental satisfaction. Bronchopulmonary dysplasia diagnosis was associated with greater adherence to recommendations (*p* < 0.001), while being underweight (*p* = 0.029) associated with more unmet recommendations.

*Conclusion*: Despite longer sleep duration, lifestyle behaviours in EP children at 2 years’ CA largely resemble those of the general population. However, poor adherence to lifestyle recommendations highlight the need for targeted interventions to reduce lifestyle-associated morbidities in EP children.

**Table:** 

**What is Known:** • *Healthy lifestyle behaviours in early childhood are essential for long-term health outcomes.* • *Extremely preterm (EP)–born children have an elevated risk of lifestyle-related morbidities. However, knowledge on lifestyle behaviours during toddlerhood in this population remains limited.*	
**What is New:** • *Characterization of lifestyle behaviours in this specific group of EP-born children at 2 years’ corrected age offers novel insights.* • *Except for longer sleep duration, lifestyle behaviours largely resemble the general population. However, over 30% of children in both EP and term populations fail to meet recommendations for 7 of the 9 lifestyle items included in the FLY-Kids tool.*

**Supplementary Information:**

The online version contains supplementary material available at 10.1007/s00431-026-06986-4.

## Introduction

Healthy lifestyle behaviours during early childhood are crucial for optimal growth and development, forming the foundation for long-term health [1, 2]. Abberant sleep patterns, excessive screentime, sedentary behaviours and poor nutrition have been linked to childhood obesity, impaired linear growth, poorer emotional regulation and potential adverse motor and neurocognitive outcomes [3–6]. Despite this, many children fail to meet (inter)national lifestyle recommendations. In the Netherlands, few toddlers meet all guidelines for nutrition, physical activity, screentime and sleep, and only 3% meet all guidelines [7–10]. Since lifestyle patterns are established during early childhood, this represents a critical window of opportunity for intervention [7, 11–13]. Equipping parents and healthcare professionals with tools to identify and promote healthy behaviours in early childhood is therefore essential [8].

Early development in children born extremely preterm (EP) is influenced by prematurity-related health issues that contribute to neonatal morbidity and may partly originate from adverse prenatal conditions [14, 15]. This developmental disruption as well as negative experiences during their neonatal intensive care unit (NICU) stay (e.g. tube feeding and endotracheal intubation) and other medical complications shape lifestyle behaviours, such as disturbed sleep patterns and feeding problems, beyond discharge [16, 17]. Sociodemographic factors and post-traumatic stress following the mother’s and child’s hospitalization may further influence parenting practices and lifestyle-related behaviours [18, 19].

Preterm-born adults face an elevated risk of developing cardiovascular (e.g. systemic hypertension, early heart failure and ischemic heart disease) and metabolic diseases (e.g. lipid disorders, obesity and diabetes mellitus) [20–24]. This can be partly explained by prematurity-related pathophysiology, but lifestyle behaviours could also contribute to cardiometabolic risks [25–28]. Nevertheless, research on early childhood lifestyle behaviours in preterm-born populations is scarce. Although healthy lifestyle promotion is acknowledged within follow-up programmes and Dutch preventive youth healthcare, EP follow-up care primarily focuses on growth and neurodevelopment, with lifestyle behaviours not structurally addressed [29–31]. This may reflect limited time, tools and knowledge regarding lifestyle behaviours in former preterm infants. Moreover, both parents and healthcare professionals may perceive the child eating, growing and developing at their own pace as major achievements after a (medically) challenging start.

Early assessment of lifestyle in EP children may guide future tailored interventions in this population. Therefore, the current study aims to (1) characterize lifestyle behaviours in EP children at 2 years’ corrected age (CA) using the Features of Lifestyle in Young Kids (FLY-Kids) screening tool [8] and to (2) compare these lifestyle behaviours to those of a reference population of similar age.

## Materials and methods

### Study design and setting

Lifestyle outcomes assessed with the FLY-Kids screening tool were compared between EP children (gestational age (GA) < 28 weeks) at 2 years’ CA and a previously published Dutch reference population of 2-year-olds (FLY-Kids study) [8]. EP children participated in the Generation P study, an ongoing multicentre prospective cohort study conducted across eight Dutch NICUs and affiliated general hospitals, examining early intestinal microbial colonization, antibiotic exposure, childhood health and neurodevelopment. Both studies were approved by local medical ethics committees (Generation P: 2014.386(A2020.190); FLY-Kids: MEC-2022–0249), and written informed consent was obtained from all parents/caregivers.

### Study population and data collection

The Generation P study includes a neonatal phase (inclusion immediately after birth) and follow-up phase at 2 years’ CA, each requiring separate informed consent. By sub-study closure (October 2025), six NICUs started follow-up recruitment. Neonatal exclusion criteria included congenital intestinal obstruction (e.g. intestinal or anal atresia, or Hirschsprung’s disease). Follow-up exclusion criteria were (1) demise of the child and (2) insufficient Dutch or English language proficiency of both parents/caregivers for survey interpretation. After informed consent, parents completed an online survey on their child’s health and lifestyle, including a modified version of the FLY-Kids screening tool (Supplementary Material 1). The FLY-Kids screening tool is described in Supplementary Material 2. It includes one item assessing parental satisfaction with the child’s lifestyle (1 = very unsatisfied to 10 = very satisfied), and nine domain-specific items covering diet (*n* = 4), eating habits (*n* = 2), physical activity (*n* = 1), screen time (*n* = 1) and sleep (*n* = 1). Colour-coded responses reflect adherence to recommendations (green = adherence; yellow, orange or red = progressively lower adherence). Six items do not include a yellow option. Data from hospital records and parental surveys were entered into Castor EDC® [32], including GA, birth weight, sex, comorbidities, age (at survey and follow-up), 2 years’ CA height and weight, parental education and country of birth, and maternal age. Contrary to the reference population, the Generation P study did not collect information on the respondents’ identity (e.g. mother, father or other caregiver).

The reference population and data collection are described previously [8]. Briefly, the reference population includes 201 children aged 1–3 years attending preventive youth healthcare consultations in the Netherlands. Before the consultation, parents completed the FLY-Kids screening tool (Supplementary Material 3) and provided basic demographic information. As GA was not collected, preterm infants may be included. No further data collection was possible, as results were anonymous. For the current study, only 2-year-olds were included in the analysis (*n* = 73).

### Statistical analysis

Child and parent characteristics were summarized as mean (standard deviation (SD)) or median (interquartile range (IQR)) for continuous variables, depending on distribution, and as counts (%) for categorical variables. Normality was assessed using histograms and Shapiro–Wilk tests. CA for the EP population was calculated as calendar age minus the days born before term. Calendar age was used for the reference population. Variable definitions are provided in Supplemental Table 1. Attrition bias was addressed by comparing baseline characteristics stratified by participation and survey response status.

For each population, FLY-Kids outcomes included parental satisfaction score, proportions of colour-coded responses across the nine lifestyle items and the proportion of children with all-green scores. A summary variable was created representing the number of items not meeting recommendations (non-green items). Group differences in baseline characteristics and outcomes between the EP and reference population were assessed using independent samples *t*-tests or Mann–Whitney *U* tests for continuous variables, and chi-square tests for categorical variables, with Fisher’s exact test or Monte Carlo simulation applied when expected cell counts were low. Significant chi-square tests were followed by post hoc analyses using adjusted standardized residuals. Associations between population characteristics and parental satisfaction score and number of non-green items were explored in the EP population using univariable and multivariable linear regression. Variables with *p* < 0.10 in univariable regression were entered into multivariable models through forward selection, with SES and parental education included as confounders. To improve comparability, final models were harmonized by including all variables retained in either final model. Missing data were handled using fully conditional specification (15 datasets, 50 iterations), including all variables from the final models. Derived anthropometric and lifestyle variables were recalculated within each imputed dataset. Estimates were pooled using Rubin’s rules and compared with complete case analyses. Finally, non-imputed data from both populations were pooled, and regression analyses were repeated for shared population characteristics using the same modelling approach. Analyses were performed in SPSS (v28.0.1.1(15)) and figures in GraphPad Prism (v10.6.0(890)).

## Results

### Population characteristics

Supplemental Fig. 1 depicts the flowchart of the inclusion process for the Generation P study. Between March 2021 and October 2025, 795 EP infants were included in the neonatal phase. By sub-study closure, 269 children reached 2 years’ CA and received a study invitation, to which parents of 239 children responded. Full informed consent was obtained from 176 children (74%), after which we received 115 completed surveys (65%), including fourteen twin-pairs. Supplemental Table 2 shows neonatal characteristics stratified by study participation and survey response status, with only birth weight differing between survey-completers and non-responders (940 vs 840 g, *p* = 0.04). Population characteristics of both cohorts are presented in Table 1, showing that anthropometric measures are significantly lower in the EP population (weight-for-height SDS: − 0.88 vs. − 0.31, *p* = 0.008). Additionally, underweight was more prevalent in the EP population (20% vs. 8%, adjusted standardized residual: 2). Sociodemographic factors are depicted in Supplemental Table 3.

**Table 1:** Characteristics of the extremely preterm and reference population

	Extremely preterm population (***n*** = 115)	Reference population (***n*** = 73)	***p***-value
Chronological age in months (IQR)	29 (4)	25 (5)	< 0.001*
Corrected age in months (IQR)	26 (4)		
Gestational age in weeks + days (IQR in days)	26 + 5 (9)	NA	
Female, ***n*** (%)	55 (48%)	35 (48%)	0.987*
Height-for-age SDS (SD) ^a^	− 0.63 (1.3)	0.33 (1.4)	< 0.001*
BMI (SD) ^a^	15.5 (1.4)	16 (1.6)	0.031*
Weight-for-height SDS (SD) ^a^	− 0.88 (1.2)	− 0.31 (1.7)	0.008*
Weight category, ***n*** (%)^a^			
Underweight	22 (20%)	6 (8%)	0.035*
Healthy weight	87 (78%)	60 (85%)	
Overweight	2 (2%)	5 (7%)	
Obesity	1 (1%)	0 (0%)	
Small for gestational age, %yes (***n***)	25 (22%)	NA	
ΔSDS weight discharge to 2 years’ CA^a^	− 0.06 (1.36)	NA	
Morbidities in first month of life, % (***n***)			
None	74 (64%)	NA	
Necrotizing enterocolitis	3 (3%)	NA	
Late-onset sepsis/meningitis	27 (24%)	NA	
Clinical sepsis	11 (9%)	NA	
Bronchopulmonary dysplasia^a^			
No	32 (31%)	NA	
Mild	36 (34%)	NA	
Moderate	9 (9%)	NA	
Severe	27 (26%)	NA	

*IQR* interquartile range, *SDS* standard deviation score, *SD* standard deviation, *CA* corrected age, *BMI* body mass index, *NA* data not available. ^a^Missing values: height and weight derivates (EP = 3, reference = 2) ΔSDS weight (EP = 6), bronchopulmonary dysplasia (EP = 10). *A *p*-value < 0.05 was considered significant

### Lifestyle behaviours in extremely preterm children

**Fig. 1:**
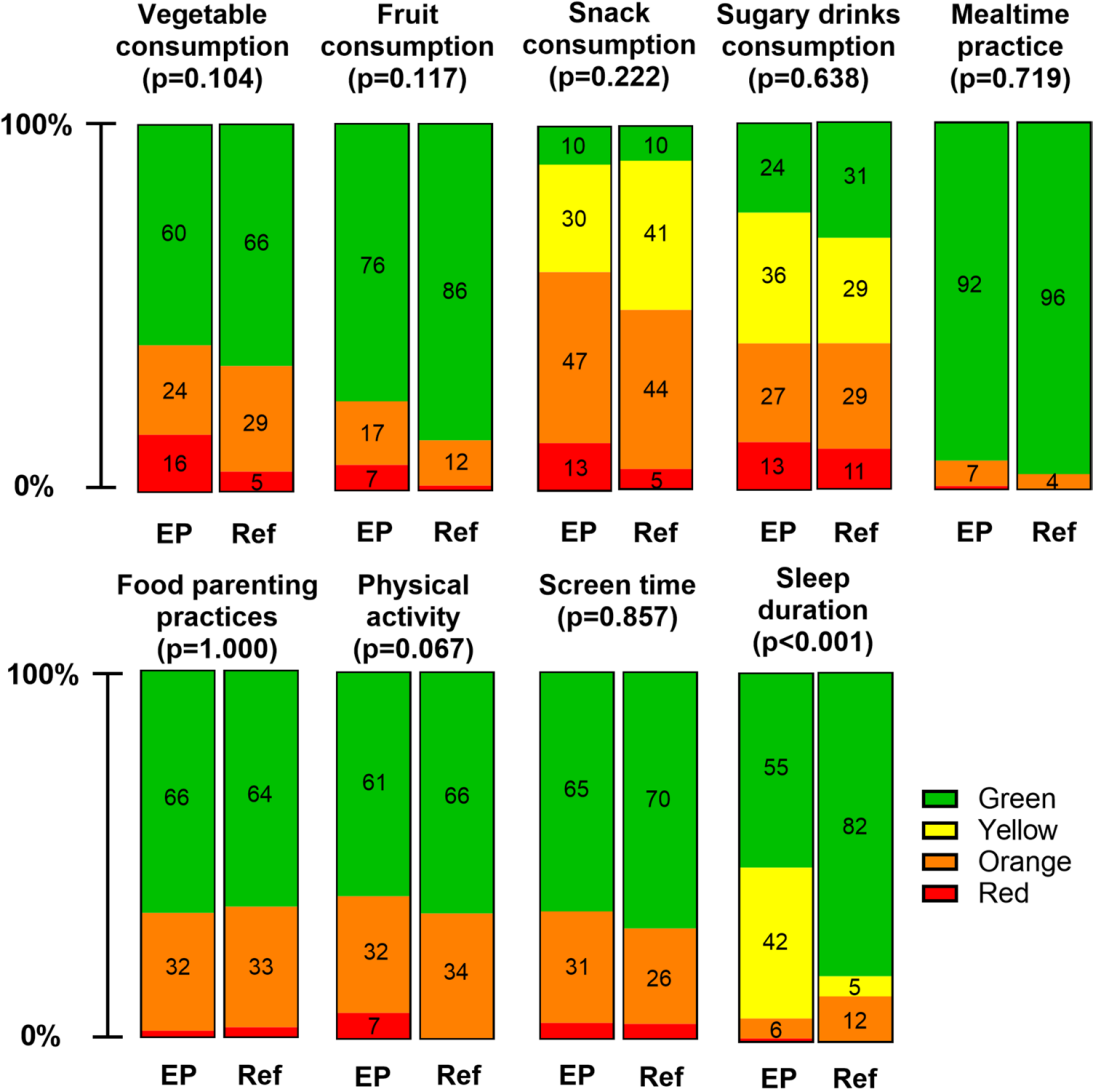
Comparison of the FLY-Kids outcomes across nine lifestyle items in the extremely preterm (EP) and reference (Ref) population. For each of the nine lifestyle items, the figure displays the proportions of children falling within each scoring category (green, yellow, orange, red), allowing for assessment of similarities and differences between the two cohorts. Green indicates adherence to national guidelines and/or recommendations, while yellow, orange or red represent progressively lower levels of adherence. For the sleep item, the distribution of scores differed significantly between populations (chi-square, *p* < 0.001). Specifically, a larger proportion of EP children sleep longer compared to the reference population: 42% sleeps 14 h or more per 24 h as represented by the yellow category (adjusted standardized residual 5.3) vs 5% of the reference population. Subsequently, only 55% of children adhere to sleep recommendations (green: 11 to 14 h per 24 h; adjusted standardized residual − 4.1) vs. 82% in the reference population, while orange (9 to 11 h per 24 h), and red (< 9 h per 24 h) are similar between populations. The distribution of scoring categories was similar across the other eight items, suggesting comparable lifestyle patterns between the populations. A *p*-value < 0.05 was considered significant

FLY-Kids outcomes for the EP population are presented in Table 2. The median parental satisfaction score was 8.5 (IQR 2; range 2–10). The mean number of non-green items was 3.92 ± 1.61. One child (0.9%) had an “all-green score”. Figure 1 shows the proportions of response categories for nine lifestyle items. The item mealtime practice was best adhered to (92% meeting recommendations). In contrast, only 10% adhered to snack consumption recommendations. In seven out of nine lifestyle items, > 30% of EP children did not meet recommendations.

**Table 2:** FLY-Kids outcomes in the extreme preterm population compared to the reference population

	Extremely preterm population (***n*** = 115)	Reference population (***n*** = 73)	***p***-value
Parental satisfaction score (median, IQR)	8.5 (2)	8 (1)	0.285
Children with all-green scores % (*n*)	0.9 (1)	2.7 (2)	0.566
Number of non-green items (mean, SD)	3.92 (1.61)	3.29 (1.57)	0.010*

*SD* standard deviation, *IQR* interquartile range. Missing values: parental satisfaction score (EP = 3), children with all-green scores (EP = 7), number of non-green items (EP = 7). *A *p*-value < 0.05 was considered significant

### Associations of population characteristics with lifestyle outcomes in extremely preterm children

Univariable associations between population characteristics and FLY-Kids outcomes (parental satisfaction score and number of non-green items) in the EP population are presented in Supplemental Table 4, and multivariable regression results are shown in Table 3. The parental satisfaction model showed that having at least one parent born outside the Netherlands and a higher SES score had a significant negative association with parental satisfaction (*B* =  − 0.88, *p* = 0.034 and *B* =  − 1.53, *p* = 0.016, respectively), after correction for parental education. The model fit varied across imputations (mean adjusted *R*^2^ = 0.09, range 0.04–0.16). Supplemental Fig. 2 depicts FLY-Kids outcomes stratified by parental country of birth (in- or outside the Netherlands).

**Table 3:** Multiple regression analysis of population predictors and parental satisfaction score and number of non-green items in the extremely preterm population after multiple imputation

	Parental satisfaction score		Number of non-green items	
	Estimate (B) (95% CI)	*p*-value	Estimate (B) (95% CI)	*p*-value
At least 1 parent born outside the Netherlands	− 0.88 (− 1.69 to 0.07)	0.034*	0.44 (− 0.38 to 1.25)	0.296
Bronchopulmonary dysplasia	− 0.52 (− 1.17 to 0.12)	0.113	− 1.11 (− 1.75 to 0.46)	< 0.001*
Socioeconomic status score	− 1.53 (− 2.76 to 0.29)	0.016*	0.42 (− 0.88 to 1.73)	0.526
Degree of urbanization (vs. not urbanized)				
Hardly	− 0.91 (− 1.85 to 0.04)	0.06	0.67 (− 0.32 to 1.66)	0.186
Moderately	− 0.07 (− 0.93 to 0.78)	0.865	0.22 (− 0.67 to 1.11)	0.628
Strongly	− 0.28 (− 1.15 to 0.59)	0.529	0.65 (− 0.27 to 1.56)	0.165
Extremely	− 0.8 (− 2 to 0.4)	0.19	1.19 (− 0.07 to 2.44)	0.064
Maternal education (vs. middle)				
Low	0.16 (− 1.39 to 1.7)	0.841	− 0.18 (− 1.75 to 1.4)	0.825
High	0.42 (− 0.24 to 1.07)	0.211	− 0.33 (− 1.02 to 0.37)	0.362
Paternal education (vs. middle)				
Low	− 0.03 (− 1.16 to 1.1)	0.959	0.03 (− 1.14 to 1.2)	0.96
High	− 0.26 (− 0.94 to 0.42)	0.452	− 0.18 (− 0.91 to 0.56)	0.642
Weight category (vs. normal weight)				
Underweight	− 0.39 (− 1.08 to 0.31)	0.276	0.82 (0.08 to 1.56)	0.029*
Overweight/obesity	− 0.72 (− 2.46 to 1.02)	0.418	− 0.57 (− 2.44 to 1.3)	0.55
Model fit	Mean *R*^2^ ≈ 0.2 and mean adjusted *R*^2^ ≈ 0.09 (range 0.03–0.17)		Mean *R*^2^ ≈ 0.27 and mean adjusted *R*^2^ ≈ 0.17 (range 0.12–0.2)	

Pooled estimates after multiple imputation. For both models, the predictors that were significantly associated to the outcome in univariate regression were included, while correcting for socioeconomic status and parental education. The predictors with a *p* < 0.1 were kept in the models. Subsequently, the models were harmonized. *A *p*-value of < 0.05 was considered significant

In the number of non-green items model, underweight at 2 years’ CA was associated with more non-green items (*B* = 0.82, *p* = 0.029, respectively). Conversely, bronchopulmonary dysplasia (BPD) was associated with fewer non-green items (*B* =  − 1.11, *p* < 0.001). The model fit was comparable across imputations (mean adjusted *R*^2^ ≈ 0.16). FLY-Kids outcomes stratified by these outcomes (underweight and BPD) are visualized in Supplemental Fig. 3 and 4, respectively.

### Except for sleep duration, lifestyle behaviours in extremely preterm children are comparable to the reference population

Lifestyle behaviours were subsequently compared between the EP and reference population. Parental satisfaction did not differ between populations (*p* = 0.285) (Table 2). Notably, only parents in the EP population reported satisfaction scores ≤ 5 (5%). The characteristics of this subgroup, with a mean of 4.67 non-green items, are displayed in Supplemental Table 5. The percentage of children with all-green scores (1% (EP) vs. 3%; *p* = 0.566) (Table 2) was comparable in both populations. The number of non-green items was significantly higher in the EP population (3.92 ± 1.6 vs. 3.29 ± 1.6; *p* = 0.010). To further explore these differences, the nine lifestyle items were compared between populations. For sleep duration, the distribution of scores differed significantly between populations (*p* < 0.001), with a higher proportion of EP children surpassing the recommended duration (≥ 14 h (yellow scores), adjusted standardized residual = 5.3). No significant differences were observed in the remaining lifestyle items (Fig. 1).

### Populations pooled: associations of shared population characteristics with FLY-Kids outcomes

Lastly, the populations were pooled to assess whether shared population characteristics were associated with FLY-Kids outcomes. Table 4 shows that parental satisfaction score was only associated with the number of non-green items (*B* =  − 0.26, *p* < 0.001). In the number of non-green items model, age at completion of FLY-Kids and being EP were associated with more non-green items (*B* = 0.07, *p* = 0.04 and *B* = 0.71, *p* = 0.003, respectively). Increased parental satisfaction lowered the number of non-green items (*B* =  − 0.36, *p* < 0.001).

**Table 4:** Multiple regression analyses examining associations between shared baseline characteristics and parental satisfaction and number of non-green items in the pooled extremely preterm and reference populations

	Parental satisfaction score		Number of non-green items	
	*B* (95% CI)	*p*-value	*B* (95% CI)	*p*-value
Extreme preterm population	0.25 (− 0.15 to 0.65)	0.21	0.71 (0.25–1.17)	0.003*
(Corrected) age at completion of FLY-kids	0.03 (− 0.04 to 0.09)	0.41	0.07 (0.01–0.15)	0.04*
Weight category (vs. normal weight)				
Underweight	− 0.18 (− 0.74 to 0.37)	0.52	0.58 (− 0.07 to 1.24)	0.08
Overweight/obesity	0.08 (− 0.83 to 0.99)	0.87	0.44 (− 0.63 to 1.51)	0.42
Satisfaction	NA	NA	− 0.36 (− 0.54 to − 0.19)	< 0.001*
Number of non-green items	− 0.26 (− 0.38 to − 0.14	< 0.001*	NA	NA
Model fit	*R*^2^ = 0.1, adjusted *R*^2^ = 0.08, *F* (5,167) 3.88, *p* = 0.002		*R*^2^ = 0.19, adjusted *R*^2^ = 0.17, *F* (5,167) 7.95, *p* = < 0.001	

*NA,* not applicable. For both models, the predictors that were significantly associated to the outcome in univariate regression were included. The predictors with a *p* < 0.1 were kept in the models. A *p*-value of < 0.05 was deemed significant. Only the number of non-green items was significantly associated to satisfaction score; hence, no other predictors are in that model. Subsequently, the models were harmonized. *A *p*-value of < 0.05 was considered significant

## Discussion

This study shows that lifestyle behaviours and parental satisfaction in EP children at 2 years’ CA are largely comparable to those of a Dutch reference population of similar age, except for sleep duration exceeding recommendations in EP children. Despite this similarity, adherence to national lifestyle recommendations was poor in both populations, underscoring the broader public health need for lifestyle improvement in all 2-year-olds, irrespective of GA.

### Lifestyle behaviours in children born extremely preterm

The finding that 2-year-old EP children exhibit lifestyle behaviours comparable to those of term-born peers is noteworthy given the medical complexity and developmental challenges associated with prematurity. While previous studies have reported feeding difficulties and reduced physical activity in EP children [16, 17, 33], we observed only longer than recommended sleep duration (≥ 14 h), consistent with earlier findings in preterm populations [34, 35]. Sleep is essential for brain maturation and neurodevelopment [36–40]; however, preterm infants are vulnerable to disrupted sleep due to brain immaturity and the NICU environment, which may adversely affect neurodevelopmental outcomes [38–40]. Increased sleep duration may therefore reflect compensatory sleep or altered regulation rather than optimal sleep quality [35, 41], although this cannot be determined from the present data. The microbiota-gut-brain axis has also been implicated in the development of sleep–wake rhythms, which is of particular interest given the altered gut microbiota development in preterm infants, though its role in this population remains unclear [42, 43]. Further research is warranted, as sleep may represent a modifiable target for optimizing neurodevelopment in this population.

Parental satisfaction scores were similar between populations, although no parent in the reference population reported a satisfaction score ≤ 5, compared to 5% in the EP population. This suggests that, despite overall high satisfaction, a subgroup of EP children experienced greater lifestyle-related challenges. Overall adherence to national recommendations was poor, with only 0.9% of EP and 3% of reference children meeting all recommendations. Adherence was lowest for snack and sugary drink consumption in both populations (10–31%), consistent with previous findings in Dutch children [8, 44]. These results are concerning given the strong associations between snack and sugary drink consumption and weight gain and cardiometabolic diseases across the lifespan [45, 46]. Given the suboptimal adherence to lifestyle recommendations, early screening, counselling and targeted lifestyle interventions may be beneficial. Addressing dietary habits through personalized counselling in early life may contribute to reduced cardiometabolic risk [47, 48]. Furthermore, evidence in EP-born children indicates that structured lifestyle interventions can reduce the likelihood of hyperlipidaemia and obesity and improve exercise capacity [49–51]. This is particularly relevant for EP-born children, who face an increased risk of lifestyle-associated diseases, but are frequently unaware of their heightened cardiometabolic risk [52]. This underscores the need for systematic attention to lifestyle behaviours in routine follow-up care to contribute to a better understanding and improved long‑term health.

### Lifestyle behaviours and the influence of parental, environmental and clinical characteristics

Within the EP population, having at least one parent born outside the Netherlands was associated with lower parental satisfaction, possibly reflecting barriers to healthcare access and challenges navigating healthcare systems among families with a migration background [53]. Although migration background negatively affected sleep duration and physical activity in previous studies [54–56], we found no association between parental country of birth and the number of non-green items. This may indicate that parental country of birth differentially influences specific lifestyle behaviours, as reported previously [57], although item-level regression analyses were not performed in this study. Higher SES was also associated with lower parental satisfaction, potentially reflecting greater awareness of developmental- and lifestyle-related norms, leading to higher expectations and lower satisfaction [58]. However, this association should be interpreted cautiously given the relatively high average SES within the EP population.

Additionally, clinical characteristics were associated with the number of non-green items. Children with BPD exhibited fewer non-green items, possibly due to increased healthcare needs and greater specialist involvement, leading to heightened attention to nutritional status and physical activity [59, 60]. Although unhealthy lifestyle behaviours are commonly associated with overweight and obesity [61], we observed an association with underweight in the EP population. Underweight EP children may experience feeding difficulties resulting in unhealthy nutritional behaviours and inadequate growth [17, 62]. These results should be interpreted in light of the low prevalence of overweight and obesity in this study (3%). However, excess adiposity in EP children may emerge later in life, as described previously [63]. Notably, 20% of EP children were underweight, consistent with previous reports, and likely reflect prematurity-related growth issues [62]. However, this finding may also be influenced by the application of Dutch growth references in a heterogenous population. Unfortunately, mid-parental height SDS were unavailable, limiting further interpretation.

### Strengths and limitations

The multicentre design of the Generation P and FLY-Kids studies ensured a cohort that was geographically representative of the Dutch population. To our knowledge, this is the first study to assess a broad spectrum of lifestyle factors in early childhood among EP children. A validated, rapid lifestyle screening tool was used, previously evaluated in a youth healthcare setting and rated as helpful and user-friendly by end-users [8]. Additionally, multiple imputation was applied to maximize the use of available data.

This study also has limitations. The questionnaire completion rate was 48%, illustrating challenges in follow-up and questionnaire-based studies. Children of non-responders had a lower birth weight; however, birth weight was not associated with FLY-Kids outcomes in univariable analyses. As follow-up data were unavailable for non-responders, response bias could only be assessed using neonatal variables, and other potential sources of bias cannot be excluded. The use of previously collected anonymous data from the FLY-Kids study limited comparisons of additional variables such as GA, SES and urbanization. Additionally, population variables were collected and defined differently between populations. The FLY-Kids study collected respondent characteristics, whereas the Generation P study collected parental information. Therefore, direct comparison of educational level was not feasible. Furthermore, regression analyses relied on a summary variable (number of non-green items), which assumes equal weight of each item and may overlook item-specific associations.

### Future research

Future research should focus on elucidating the mechanisms and implications of prolonged sleep in EP children. Furthermore, the observed associations between baseline characteristics and FLY-Kids outcomes should be further explored in larger EP populations before strong conclusions can be drawn regarding lifestyle risk stratification. Additionally, future research should investigate implementing the FLY-Kids lifestyle screening concept within routine follow-up care for EP children. This concept includes completing the questionnaire prior to the visit, indicating which lifestyle items parents wish to discuss, and providing notes for healthcare professionals. After completion, parents receive personalized advice, which can be further explored during the visit.

## Conclusion

In conclusion, although lifestyle behaviours in EP children at 2 years’ CA closely resembled those of the reference population, except for longer sleep duration, and parental satisfaction was high, adherence to lifestyle recommendations in all 2-year-olds is poor. Opportunities for improving lifestyle behaviours may arise before this age, indicating that early counselling and targeted interventions could be beneficial. In this context, the FLY-Kids screening tool may help identify lifestyle recommendations adherence and support early risk stratification. Future interventions should be culturally sensitive and tailored to the specific needs of families, with special attention to high-risk subgroups such as children who are underweight. Addressing these challenges early may improve lifestyle behaviours and help reduce the long-term risk of lifestyle-related diseases in this vulnerable population.

## Supplementary Information

Below is the link to the electronic supplementary material.

ESM 1 Supplementary Material 1 (DOCX 863 KB)

## Data Availability

The data used in this study are available upon reasonable request from the corresponding author.

## Abbreviations

BPD: Bronchopulmonary dysplasia
CA: Corrected age
EP: Extremely preterm
FLY-Kids: Features of Lifestyle in Young Kids
GA: Gestational age
IQR: Interquartile range
NICU: Neonatal intensive care unit
SD: Standard deviation
SDS: Standard deviation scores
SES: Socioeconomic status
