# Supplementary material for: Lifestyle behaviours in children born extremely preterm at 2 years: a comparison to a Dutch reference population
Source: Eur J Pediatr. 2026 Apr 29;185(5):323. doi: 10.1007/s00431-026-06986-4 (PMC13128773; doi:10.1007/s00431-026-06986-4)
Supplement: Supplementary file 1 — Supplementary Material 1 (DOCX 863 KB) [file 431_2026_6986_MOESM1_ESM.docx]

**Supplemental Information to the following manuscript:**

**Lifestyle Behaviours in Children Born Extremely Preterm at Two Years: A Comparison to a Dutch Reference Population**

N.M. Frerichs^1,2,3^*, A.M. le Clercq^4,5^*, M.C.J. Kooij^5^, J.J.A. Krijger^5^, A.J. van Wesemael^1,2,3^, R.R. de Kroon^1,2,3^, C.H.P. van den Akker^2,3,6^, M.M.A. Raets^7^, E. J. d’Haens^8^, W.P. de Boode^9^, E. Roze^4^, J.B. van Goudoever^2,6^, A.G. Leemhuis^2,6^, B.J. Vlieg-Boerstra^10^, E.G. van Mil^11,12^, A. Kindermann^1^, K.F.M. Joosten^5^, H.J. Niemarkt^13^**, T.G.J. de Meij^1^**.

*Contributed equally as first authors;

**Contributed equally as last authors.

**Affiliations:**

^1^Department of Pediatric Gastroenterology, Emma Children’s Hospital, Amsterdam UMC, Amsterdam, The Netherlands; ^2^Amsterdam Gastroenterology Endocrinology Metabolism Research Institute, Amsterdam, The Netherlands; ^3^Amsterdam Reproduction and Development Research Institute, Amsterdam, The Netherlands; ^4^Department of Neonatal and Pediatric Intensive Care, Division of Neonatology, Erasmus Medical Center, Rotterdam, the Netherlands; ^5^Department of Neonatal and Pediatric Intensive Care, Division of Pediatric Intensive Care, Erasmus Medical Center, Rotterdam, the Netherlands; ^6^Department of Pediatrics - Neonatology, Amsterdam UMC, Emma Children's Hospital, University of Amsterdam, Amsterdam, The Netherlands; ^7^Division of Neonatology, Department of Pediatrics, Maastricht University Medical Center+, MosaKids Children’s Hospital, Maastricht, The Netherlands; ^8^Department of Neonatology, Isala Klinieken, Zwolle, the Netherlands; ^9^Division of Neonatology, Department of Pediatrics, Radboud University Medical Center, Radboud Institute for Health Sciences, Amalia Children's Hospital, Nijmegen, Netherlands; ^10^Department of Pediatrics, OLVG Hospital, Amsterdam, The Netherlands; ^11^Department of Pediatrics, Jeroen Bosch Hospital, 's-Hertogenbosch, The Netherlands; ^12^Maastricht University, Maastricht, The Netherlands; ^13^Neonatal Intensive Care Unit, Máxima Medical Center, Veldhoven, The Netherlands.

**Supplementary Material 1 – FLY-kids questionnaire Generation P study**

1. How satisfied are you with your child’s lifestyle (diet, physical activity, screen time, sleep)?
   Response options presented on a slider bar:
   - 0 (Treated as missing, indicating that the slider was not moved)
   - 1 (Not at all satisfied)
   - 2 - 9
   - 10 (Very satisfied)
2. How many vegetables does your child eat per day? *Consider all vegetables your child eats, including snacks like cucumber or carrots.*

Response options:

- My child does not eat vegetables
- Less than half a serving spoon per day
- Half to one serving spoon per day
- One serving spoon or more per day

1. How many days a week does your child eat fruit?

Response options:

- My child does not eat fruit
- Less than 4 days per week
- 4 to 6 days per week
- Every day

1. How many sugar-sweetened beverages does your child drink per day? *Consider for example, soft drinks, fruit juices, thick juice, lemonade, and milk drinks with sugar such as chocolate milk and yoghurt drinks.*

Response options:

- No drinks containing sugar
- Less than 1 glass or juice box per day
- 1 glass or juice box per day
- 2 or more glasses or juice boxes per day

1. How many snacks does your child eat per day? Consider for example, cookies, candy, crisps, and cake.

Response options:

- No snacks
- Less than 1 snack per day
- 1 snack per day
- 2 snacks or more per day

1. How often does your child eat his/her meals at the dining table?

Response options:

- Almost never
- Occasionally
- Almost always

1. How often do you give your child something to eat to comfort or reward him/her?

Response options:

- Almost never
- Occasionally
- Almost always

1. How much time is your child physically active per day? *Consider for example, active (outdoor) playing, cycling, crawling, playing with a ball, moving to music, running, and jumping.*

Response options:

- Less than 1.5 hours per day
- 1.5 to 3 hours per day
- 3 hours or more per day
- I do not know
  - Treated as missing

1. How much time does your child spend using electronic screens per day? *Consider for example, TV, computer, mobile phone, tablet.*

Response options:

- 0 to 1 hour per day
- 1 to 2 hours per day
- 2 hours of more per day

1. How many hours per day (per 24 hours) does your child sleep? *Including daytime naps.*

Response options: 0 to 24 hours

Categorization of response options:

- <=8.99
- 9.00-10.99
- 11.00-13.99
- =>14
- 0
  - Treated as missing

**Supplementary Material 2 – Supplemental Methods**

**FLY-Kids screening tool**

To support healthcare professionals in assessing lifestyle behaviors as well as in discussing and supporting a healthy lifestyle with parents, the Features of Lifestyle in Young Kids (FLY-Kids) screening tool was developed for children aged 1 to 3 years [1]. It provides a brief, parent-administered assessment regarding key lifestyle domains such as healthy and unhealthy food intake, eating habits, and other lifestyle factors. In a reference population of toddlers attending preventive youth healthcare, FLY-Kids has been shown to be both helpful and user-friendly, with an evaluation study demonstrating usability, feasibility, and preliminary effects on initiating lifestyle discussions during preventive youth healthcare consultations [1]. The FLY-Kids is available in the Dutch and English language.

The screening tool includes 10-items in total. The first item asks parent(s) and/or caregiver(s) to grade their satisfaction with their child’s lifestyle on a scale from 1 (very unsatisfied) to 10 (very satisfied), followed by nine items including vegetable, fruit, snack and sugar-sweetened beverage intake, eating habits (eating at the dinning table and using food as a reward), and hours of physical activity, screen time, and sleep. The answers to the nine lifestyle items are categorized by colour which indicate the extent to which the national guidelines and/or recommendations are met. Green indicates perfect adherence, while yellow, orange, or red represent progressive lower levels of adherence. Six items exclude yellow as a response option.

The original paper version of the 2-year FLY-kids screening tool can be found in **Supplementary Material 3.** In the reference population, parent(s) and/or caregiver(s) that were present during the youth healthcare appointment were asked to complete a paper version of FLY-kids questionnaire before consultation started. The outcomes were discussed during the consultation based on the color of the item scores. If an item scored green, no adjustments on lifestyle were needed, if it scored yellow then it was discussed as an item that needs attention, and if the item scored orange or red, possible lifestyle adjustments were discussed with the parent(s) and/or caregiver(s).

A modified version of the FLY-Kids questionnaire for 2-year-old children was embedded in the Generation P online parental survey (Supplementary Material 1). Following the standard 2-year corrected age follow-up visit, parents were provided with a survey link by email or via a personalised QR code. Although parental information is collected as part of the Generation P study, details regarding the identity of the person who completed the survey were not asked. Additionally, no feedback on their child’s lifestyle was provided to parent(s) and/or caregiver(s) after completion of the survey. The exact items with response options and coding (including the coding of missing values) are provided in Supplementary Material 1. Modifications of the FLY-Kids tool include: (1) no images were shown with the questions, (2) the items “Vegetables” and “Fruit” included an extra answering option (“My child does not eat vegetables/fruit”), (3) the item “Physical Activity” included the answering option “I do not know”, and (4) the item sleep was measured numerically and later categorized. The items were distributed across the survey, for example the items “Vegetables” and “Fruit” were asked in the form regarding feeding while the item “Sleep” was asked in the form on lifestyle, and thus not all administered in the order presented in **Supplementary Material** **1**.

**Linear regression models and imputation of missing data**

The influence of independent baseline variables on parental satisfaction score and the number non-green items was explored in the EP population by univariable and multiple linear regression analyses. The multiple regression analyses were performed using the forward selection method to investigate associations while minimizing collinearity. Candidate baseline predictors with a p-value of <0.1 in univariable linear regression analysis were tested in the multiple regression models with parental satisfaction and number of non-green items as the dependent variables. Categorical variables were evaluated and removed as a whole (k−1 dummy variables). Only variables with a p-value of <0.1 were included in the final models. The models were adjusted for SES score and parental education as these are considered potential confounding factors for lifestyle behaviours [2]. To improve comparability between outcomes and account for potentially shared confounding structures, the final models were subsequently harmonized by including all variables retained in either outcome model. The independent variables are depicted in **Supplemental Table 3**. Prior to conducting the linear regression analyses, the missing values in the EP population were addressed using multiple imputation. The missing at random assumption was assessed by comparing baseline characteristics between participants with complete and incomplete data. All variables included in the final regression models were entered into the imputation model. Imputation was performed using a fully conditional specification approach with Markov Chain Monte Carlo estimation, predictive mean matching for continuous variables and logistic regression for categorical variables. A total of 15 imputed datasets were generated over 50 iterative cycles. Following imputation, variables derived from discharge weight (ΔSDS weight), and height and weight at 2-years’ CA (height-for-age SDS, weight-for-height SDS, BMI, BMI SDS, and weight category), and the item scores for physical activity and sleep (number of non-green items) were recalculated separately within each of the 15 imputed datasets. Each completed dataset was subsequently analysed, and results were pooled using Rubin’s rules to obtain pooled estimates. The pooled estimates were compared to the complete case analysis. The pooled estimates in both models were consistent across imputations, with variability only observed in low-frequent categories (maternal and paternal education). Model assumptions were evaluated in a representative imputed dataset. Linearity and homoscedasticity were assessed using residual plots, and normality of residuals was evaluated using Q–Q plots. Multicollinearity was examined using variance inflation factors. No major violations of model assumptions were observed. Finally, the non-imputed datasets from the two cohorts were pooled, and univariate and multiple linear regression analyses were repeated for the shared populations characteristics (age at completion of FLY-kids, biological sex, BMI, height-for-age SDS, weight-for-height SDS, and weight category) using the forward selection method.

**Supplementary Material 3 – FLY-kids questionnaire reference population [1]**

**FLY-Kids Please tick the box of your choice for each question**

| **1. How satisfied are you with your child’s lifestyle?**  Circle a number between 1 and 10 | Satisfaction  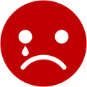  1 2 3 4 5 6 7 8 9 10 |
| --- | --- |
| **2. How many vegetables does your child eat per day?**  Consider all vegetables your child consumes, including, for example, cucumber in between meals  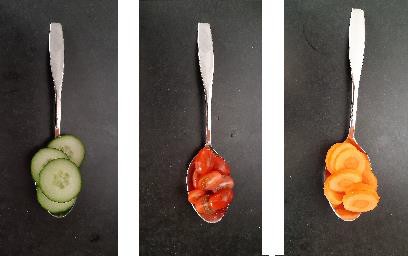  Examples of 1 serving spoon of vegetables | - Less than half a serving spoon a day - Half to 1 serving spoon a day - 1 serving spoon or more a day |
| **3. How many days a week does your child eat fruit?**  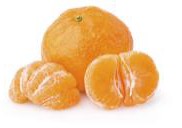 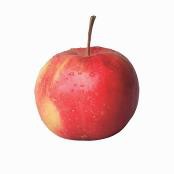 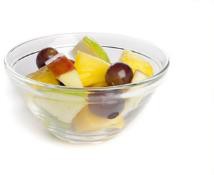 | - Less than 4 days a week - 4 to 6 days a week - Every day |
| **4. How many sugar-sweetened beverages does your child drink per day?**  Consider, for example, soft drinks, fruit juice, thick juice, lemonade, and milk drinks with sugar, such as chocolate milk and yoghurt drink.  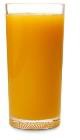 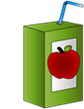 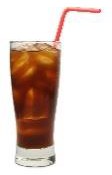 | - None - Less than 1 glass or juice box a day - 1 glass or juice box a day - 2 glasses or juice boxes or more a day |
| **5. How many snacks does your child eat per day?** Consider, for example, cookies, candy, crisps, and cake. 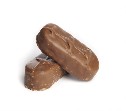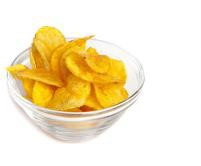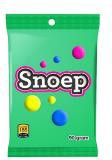 | - None - Less than 1 snack a day - 1 snack a day - 2 snacks or more a day |
| **6. How often does your child eat his/her meals at the dining table?**  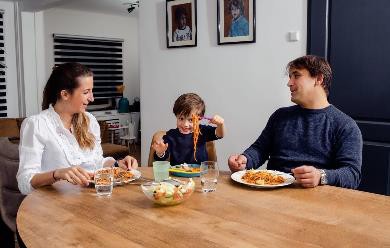 | - Almost never - Occasionally - Almost always |
| **7. How often do you give your child something to eat to comfort or reward him/her?**  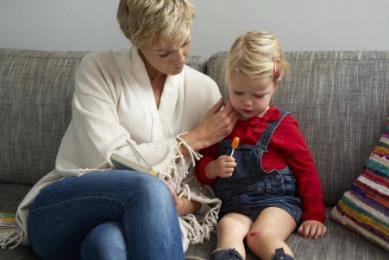 | - Almost never - Occasionally - Almost always |
| **8. How much time is your child physically active per day?**  Consider, for example, active (outdoor)playing, cycling, crawling, playing with a ball, moving to music, running, and jumping.  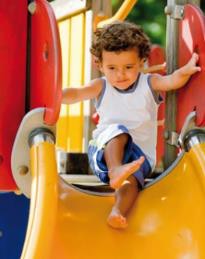 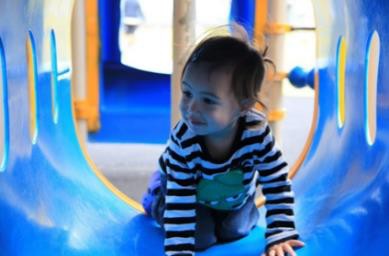 | - Less than 1.5 hours a day - 1.5 to 3 hours a day - 3 hours or more a day |
| **9. How much time does your child spend using electronic screens per day?**  Consider, for example, TV, computer, mobile phone and tablet.  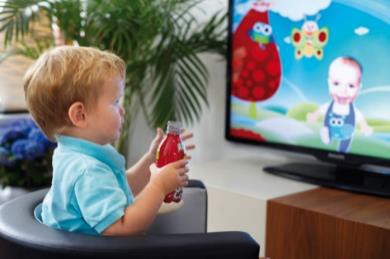 | - 0 to 1 hour a day - 1 to 2 hours a day - 2 hours or more a day |
| **10. How much time does your child sleep per 24 hours?**  Also include naps during daytime.  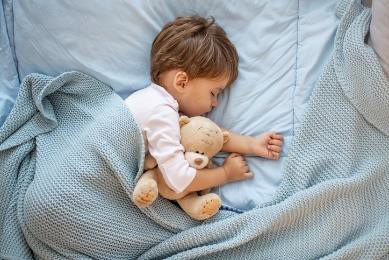 | - Less than 9 hours per 24 hours - 9 to 11 hours per 24 hours - 11 to 14 hours per 24 hours - More than 14 hours per 24 hours |

**Supplemental Tables**

| Supplemental Table 1: Variable definitions | |
| --- | --- |
| Variable: | **Definitions:** |
| Height-for-age SDS | Calculated with Growth Analyser RCT (v4.0.28) using Dutch reference values adjusted for prematurity [3]. |
| Weight-for-height SDS |  |
| BMI |  |
| BMI SDS |  |
| Weight classification | Weight status was classified following the national guidelines for underweight, overweight, and obesity. Weight-for-height SDS were used for the classification of underweight (<-2 SDS) and age- and sex-specific BMI cut-offs according to the revised International Obesity Task Force BMI cut-offs were used to classify overweight and obesity [4]. |
| Small for gestational age^a^ | Calculated using the Fenton 2025 Preterm Growth Chart [5, 6]. |
| Discharge weight SDS^a^ | Calculated with the Fenton 2025 Preterm Growth Chart and PediTools using NICU discharge age and weight. |
| Delta weight SDS (ΔSDS weight)^a^ | Calculated from NICU discharge weight SDS to weight-for-age SDS at 2-year CA follow-up (ΔSDS weight = SDS_24months_​−SDS_discharge_). |
| Parental country of birth^a^ | Categorized as both parents born in the Netherlands or at least one parent born outside the Netherlands [7]. |
| Respondent country of birth^b^ | Categorized as born in the Netherlands or born outside the Netherlands [7]. |
| Parental educational level^a^ | Categorized into three levels (low, middle, and high) based on the highest completed education. Educational categories were derived from the Dutch education system and aligned with the International Standard Classification of Education (ISCED 2011). Low education corresponds to ISCED level 0-2, middle to ISCED levels 3-4, and high to ISCED levels 5-8. |
| Respondent educational level^b^ |  |
| Degree of urbanization^a^ | Categorized as 1) not, (2) hardly, (3) moderately, (4) strongly, and (5) extremely urbanised based on the postal code using “Check je Plek” from the Dutch ministry of Infrastructure and Water Management [8]. |
| Socioeconomic status (SES) score^a^ | Socioeconomic status (SES) score was obtained based on the postal code using the SES-WOA (Socioeconomic status based on wealth, education, and employment) score from Statistics Netherlands (CBS) [9]. |
| Co-morbidities in the first month of life^a^ | Culture proven sepsis was defined as (1) a positive blood culture, (2) generalized signs of infection, and (3) intention to treat with antibiotics for 5 consecutive days. Clinical sepsis was defined as (1) generalized signs of infection, (2) a C-reactive protein (CRP) >10mg/L, and (3) intention to treat with antibiotics for 5 consecutive days. Necrotizing enterocolitis was defined as Bell’s Stage ≥2A. Meningitis was defined as (1) a positive cerebrospinal fluid culture and/or CSF WBC count > 30/µL, (2) generalized signs of infection, and (3) intention to treat with antibiotics for 7 consecutive days. Bronchopulmonary dysplasia (BPD) status was extracted from patient files and categorized as mild, moderate or severe BPD [10]. |
| ^a^Only collected in the extreme preterm population. ^b^Only collected in the reference population.  Abbreviations: SDS, standard deviation scores; BMI, body mass index; NICU, neonatal intensive care unit. | |

| Supplemental Table 2: Comparison of neonatal characteristics of eligible children by participation and survey response status in the extremely preterm population. | | | |
| --- | --- | --- | --- |
|  | **Study invitation send (n=269)** | | **p-value** |
|  | Responder (n=239) | Non-responder (n=30) |  |
| Female, n (%) | 114 (48%) | 11 (37%) | 0.25 |
| Birth weight in grams, median (IQR) | 880 (283) | 935 (230) | 0.45 |
| Gestational age, weeks+days (IQR In days) | 26+4 (10) | 26+3 (15) | 0.51 |
|  | **Responders to study invitation (n=239)** | |  |
|  | Included (n=176) | Excluded (n=63) |  |
| Female, n (%) | 84 (47%) | 30 (49%) | 0.88 |
| Birth weight in grams, median (IQR) | 850 (353) | 910 (259) | 0.16 |
| Gestational age, weeks+days (IQR in days) | 26+4 (12) | 26+4(10) | 0.6 |
|  | **Included at 2-years corrected age (n=176)** | |  |
|  | Survey response (n=115) | No survey (n=61) |  |
| Female, n (%) | 55 (48%) | 29 (48%) | 1.00 |
| Birth weight in grams, median (IQR) | 940 (280) | 840 (263) | 0.04* |
| Gestational age, weeks+days (IQR in days) | 26+5 (9) | 26+3 (12) | 0.1 |
| Abbreviations: IQR, interquartile range. *A p-value<0.05 was considered significant. | | | |

| Supplemental Table 3. Sociodemographic characteristics of the extremely preterm and reference population. | | |
| --- | --- | --- |
|  | **Extremely preterm population (n=115)** | **Reference population (n=73)** |
| Maternal age in years (IQR) ^a^ | 34 (7) | NA |
| Respondent age in years (IQR) | NA | 36 (5) |
| Parental country of birth % (n) ^a^ |  |  |
| Both born in the Netherlands | 74 (77%) | NA |
| At least one parent born outside the Netherlands | 22 (23%) | NA |
| Respondent country of birth % (n) |  |  |
| In the Netherlands | NA | 60 (82%) |
| Outside the Netherlands | NA | 13 (18%) |
| Maternal education % (n) ^a^ |  |  |
| Low | 2 (2%) | NA |
| Middle | 49 (47%) | NA |
| High | 53 (51%) | NA |
| Paternal education % (n) ^a^ |  |  |
| Low | 7 (7%) | NA |
| Middle | 46 (47%) | NA |
| High | 45 (46%) | NA |
| Respondent education % (n) |  |  |
| Low | NA | 4 (6%) |
| Middle | NA | 15 (21%) |
| High | NA | 53 (73%) |
| Socioeconomic status (IQR) | 0.175 (0.39) | NA |
| Degree of urbanisation % (n) |  |  |
| Not urbanised | 24 (21%) | NA |
| Hardly urbanised | 15 (13%) | NA |
| Moderately urbanised | 26 (23%) | NA |
| Strongly urbanised | 37 (32%) | NA |
| Very strongly urbanised | 13 (11%) | NA |
| Abbreviations: IQR, interquartile range; NA, data not available. ^a^Missing values: Parental country of birth (EP: 19), maternal age (EP:1), maternal education (EP: 11), paternal education (EP: 17). Degree of urbanisation and socioeconomic status were based on postal code at 2-years’ corrected age. | | |

| Supplemental Table 4. Univariable associations between baseline predictors and parental satisfaction score and number of non-green items. Pooled estimates after multiple imputation and complete case analysis. | | | | | | | | |
| --- | --- | --- | --- | --- | --- | --- | --- | --- |
|  | **Parental satisfaction score** | | | | **Number of non-green items** | | | |
|  | **Pooled estimates** | | **Complete case analysis** | | **Pooled estimates** | | **Complete case analysis** | |
|  | **Estimate (B) (95% CI)** | **p-value** | **Estimate (B) (95% CI)** | **p-value** | **Estimate (B) (95% CI)** | **p-value** | **Estimate (B) (95% CI)** | **p-value** |
| Age at completion of FLY-kids | 0.02 (-0.06-0.11) | 0.59 | 0.024 (-0.063-0.112) | 0.59 | 0.05 (-0.05-0.14) | 0.32 | 0.06 (-0.04-0.15) | 0.25 |
| Gestational age | 0.01 (-0.04-0.05) | 0.87 | 0.005 (-0.038-0.048) | 0.82 | 0.04 (-0.01-0.08) | 0.11 | 0.04 (-0.01-0.09) | 0.1 |
| Biological sex | 0.14 (-0.41-0.69) | 0.62 | 0.128 (-0.433-0.689) | 0.65 | 0.29 (-0.31-0.88) | 0.35 | 0.14 (-0.48-0.76) | 0.66 |
| Height-for-age SDS | 0.06 (-0.17-0.28) | 0.63 | 0.055 (-0.171-0.281) | 0.63 | -0.04 (-0.23-0.21) | 0.77 | -0.07 (-0.32-0.18) | 0.57 |
| ΔSDS weight | 0.06 (-0.15-0.27) | 0.57 | 0.076 (-0.142-0.294) | 0.49 | -0.02 (-0.25-0.21) | 0.87 | -0.01 (-0.24-0.22) | 0.93 |
| BMI | 0.09 (-0.11-0.29) | 0.37 | 0.078 (-0.130-0.286) | 0.46 | -0.12 (-0.34-0.1) | 0.3 | -0.1 (-0.32-0.13) | 0.4 |
| Weight-for-height SDS | 0.12 (-0.1-0.35) | 0.29 | 0.112 (-0.126-0.350) | 0.35 | -0.18 (-0.43-0.08) | 0.17 | -0.15 (-0.41-0.11) | 0.24 |
| Weight category (vs. normal weight) |  |  |  |  |  |  |  |  |
| Underweight | -0.4 (-1.12-0.26) | 0.26 | -0.423 (-1.147-0.301) | 0.25 | 0.79 (0.02-1.55) | 0.045* | 0.78 (-0.01-1.57) | 0.05 |
| Overweight/Obesity | -0.28 (-0.34-0.74) | 0.74 | -0.471 (-2.216-1.275) | 0.59 | -0.69 (-2.48-1.1) | 0.5 | -0.48 (-2.34-1.38) | 0.61 |
| SGA (vs. AGA/LGA) | 0.03 (-0.63-0.69) | 0.93 | 0.028 (-0.636-0.712) | 0.91 | 0.26 (-0.46-0.99) | 0.48 | 0.28 (-0.49-1.04) | 0.48 |
| BPD (vs. no BPD) | -0.42 (-1.07-0.23) | 0.21 | -0.362 (-0.939-0.216) | 0.22 | -1.14 (-1.76-(-)0.51) | <0.001* | -1.17 (-1.82-(-)0.51) | 0.001* |
| Morbidities (first month of life) (vs. none) | -0.13 (-0.7-0.44) | 0.65 | -0.133 (-0.719-0.452) | 0.65 | 0.08 (-0.54-0.71) | 0.79 | 0.13 (-0.52-0.78) | 0.7 |
| Maternal age | -0.02 (-0.08-0.04) | 0.57 | -0.017 (-0.077-0.041) | 0.58 | -0.02 (-0.09-0.04) | 0.5 | -0.02 (-0.09-0.05) | 0.56 |
| Maternal education (vs. middle) |  |  |  |  |  |  |  |  |
| Low | 0.17 (-1.48-1.81) | 0.84 | 0.987 (-1.619-3.593) | 0.45 | -0.34 (-2.41-1.73) | 0.75 | -2.61 (-5.36-0.15) | 0.06 |
| High | 0.13 (-0.49-0.75) | 0.68 | 0.218 (-0.484-0.919) | 0.54 | -0.4 (-1.07-0.28) | 0.25 | -0.21 (-0.96-0.54) | 0.58 |
| Paternal education (vs. middle) |  |  |  |  |  |  |  |  |
| Low | -0.24 (-1.27-0.8) | 0.65 | -0.941 (-2.404-0.522) | 0.21 | 0.12 (-1.08-1.31) | 0.85 | 1.5 (-0.15-3.16) | 0.07 |
| High | -0.25 (-0.74-0.46) | 0.46 | -0.344 (-1.059-0.371) | 0.34 | -0.13 (-0.85-0.59) | 0.73 | -0.2 (-0.96-0.56) | 0.61 |
| Parental country of birth | -0.75 (-1.48-(-)0.02 | 0.04* | -0.890 (-1.532-(-)0.248 | 0.01* | 0.8 (0.07-1.53) | 0.03* | 0.98 (0.19-1.76) | 0.02* |
| Socioeconomic status score | -0.67 (-1.7-0.37) | 0.21 | -0.694 (-1.747-0.359) | 0.19 | -0.52 (-1.65-0.62) | 0.37 | -0.46 (-1.63-0.7) | 0.44 |
| Degree of urbanization (vs. not urbanised) |  |  |  |  |  |  |  |  |
| Hardly urbanised | -0.97 (-1.93-(-)0.01) | 0.048* | -1.032 (-2,035-(-)0.030) | 0.04* | 0.67 (-0.36-1.7) | 0.2 | 0.78 (-0.29-1.85) | 0.15 |
| Moderately urbanised | -0.23 (-1.06-0.59) | 0.58 | -0.241 (-1.091-0.608) | 0.575 | 0.2 (-0.69-1.08) | 0.66 | 0.13 (-0.8-1.06) | 0.78 |
| Strongly urbanised | -0.35 (-1.12-0.41) | 0.37 | -0.359 (-1.148-0.431) | 0.37 | 0.58 (-0.24-1.4) | 0.17 | 0.57 (-0.27-1.4) | 0.18 |
| Extremely urbanised | -0.89 (-1.89-0.11) | 0.08 | -0.895 (-1.921-0.131) | 0.087 | 1.53 (0.46-2.61) | 0.005* | 1.48 (0.36-2.6) | 0.01* |
| SDS, standard deviation score; BMI, body mass index; SGA, small for gestational age; BPD, bronchopulmonary dysplasia. *A p-value<0.05 was considered significant. | | | | | | | | |

| Supplemental Table 5: Characteristics of extremely preterm children with a <6 vs. ≥6 parental satisfaction score | | | |
| --- | --- | --- | --- |
|  | **Extremely preterm population (n=112)^b^** | | **p-value** |
|  | Parental satisfaction <6 (n=6) | Parental satisfaction ≥6 (n=106) |  |
| Corrected age in months (IQR) | 26 (1.5) | 26.5 (4) | 0.322 |
| Gestational age in weeks+days (IQR in days) | 183 (20) | 187 (8) | 0.282 |
| Female % (n) | 4 (67%) | 55 (52%) | 0.434 |
| Parental satisfaction score (IQR) | 5 (1.5) | 9 (2) | <0.001* |
| Number of non-green items (SD) | 4.67 (1.8) | 3.92 (1.6) | 0.274 |
| Height-for-age SDS (SD)^a^ | -0.19 (1.2) | -0.66 (1.3) | 0.382 |
| BMI (SD)^a^ | 15.7 (2.6) | 15.5 (1.3) | 0.845 |
| Weight-for-height SDS (SD)^a^ | -0.69 (1.9) | -0.88 (1.2) | 0.719 |
| ΔSDS weight (Discharge to 24 months) (SD)^a^ | 0.23 (1.3) | -0.03 (1.3) | 0.753 |
| Small for gestational age, %yes (n) | 0 (0%) | 25 (24%) | 0.335 |
| Bronchopulmonary dysplasia, yes % (n)^a^ | 4 (100%) | 67 (68%) | 0.311 |
| Maternal age in years (IQR) | 33 (5) | 34 (7) | 0.680 |
| Parental country of birth % (n)^a^ |  |  | 0.01* |
| Both in the Netherlands | 1 (20%) | 71 (80%) |  |
| At least one parent outside the Netherlands | 4 (80%) | 18 (20%) |  |
| Maternal education % (n)^a^ |  |  | 0.716 |
| Low | 0 (0%) | 2 (2.1%) |  |
| Middle | 2 (33.3%) | 45 (46.9%) |  |
| High | 4 (66.7%) | 4 (51%) |  |
| Paternal education % (n)^a^ |  |  | 0.300 |
| Low | 1 (16.7%) | 6 (6.7%) |  |
| Middle | 1 (16.7%) | 43 (47.8%) |  |
| High | 4 (66.7%) | 41 (45.6%) |  |
| Social economic status score (IQR) | 0.205 (0.35) | 0.165 (0.4) | 0.570 |
| Degree of residential urbanization % (n) |  |  | 0.619 |
| Not urban | 1 (16.7%) | 21 (20%) |  |
| Slightly urban | 2 (33.3%) | 12 (11%) |  |
| Moderately urban | 1 (16.7%) | 25 (24%) |  |
| Strongly urban | 1 (16.7%) | 36 (34%) |  |
| Very strongly urban | 1 (16.7%) | 12 (11%) |  |
| Abbreviations: IQR, interquartile range; SDS, standard deviation score; SD, standard deviation; BMI, body mass index. ^a^Missing values: Parental country of birth: 18, maternal education: 10, paternal education: 16, anthropometrics: 3, ΔSDS weight: 6, bronchopulmonary dysplasia: 10. ^b^Parental satisfaction score was missing in 3 children. *A p-value<0.05 was considered significant. | | | |

**Supplemental Figures**


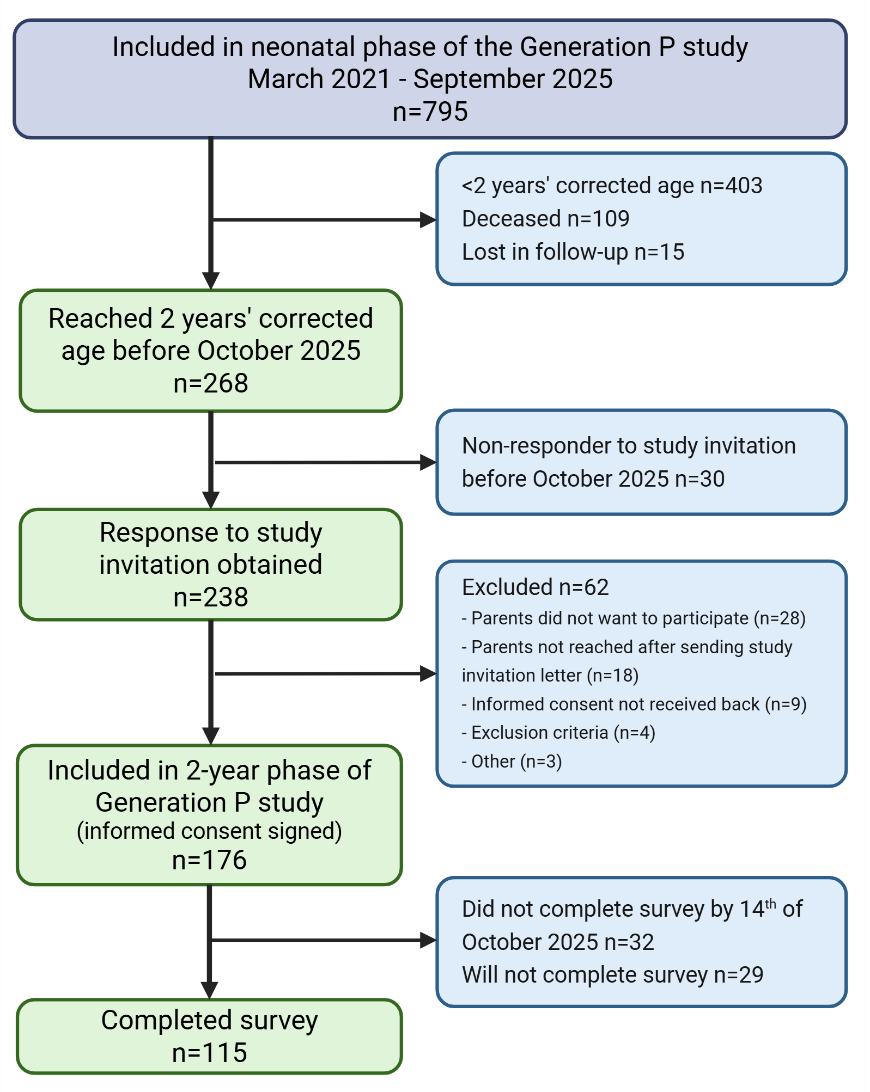


**Supplemental Fig. 1** **Flowchart of the inclusion process in the Generation P study** Created in BioRender. Amsterdam UMC, E. (2026) <https://BioRender.com/5f6wp6t>


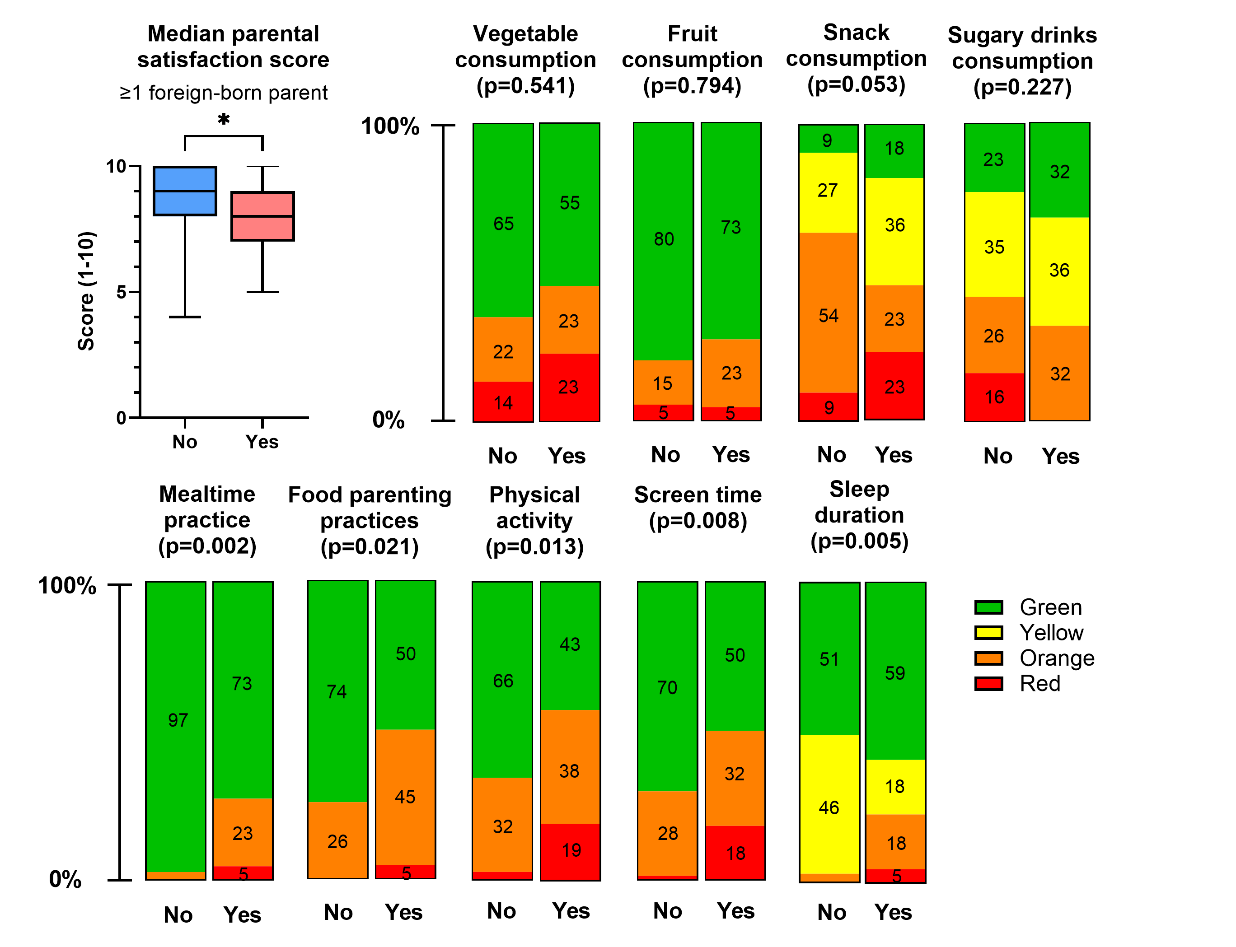


**Supplemental Fig. 2 Distribution of FLY-Kids lifestyle scores within the extremely preterm population, comparing children with ≥1 foreign-born parent (Yes) to children whose parents are both born in The Netherlands (No)** This figure presents the median parental satisfaction score within each group and the proportion of children in each scoring category (green, yellow, orange, red) across the nine lifestyle items. Colour-coding reflects adherence to age-specific lifestyle recommendations, with green indicating adherence and yellow, orange, and red indicating progressively lower adherence. Parental satisfaction was significantly lower in the ≥1 foreign-born parent subgroup. Additionally, the distribution of mealtime practices (eating at the dinner table), food parenting practices (comforting/rewarding children with food), physical activity, screen time, and sleep duration differed significantly between children with and without ≥1 foreign-born parent. Specifically, the distribution shifted towards more orange and/or red categories in the ≥1 foreign-born parent subgroup, reflecting poorer adherence to recommendations for these items.


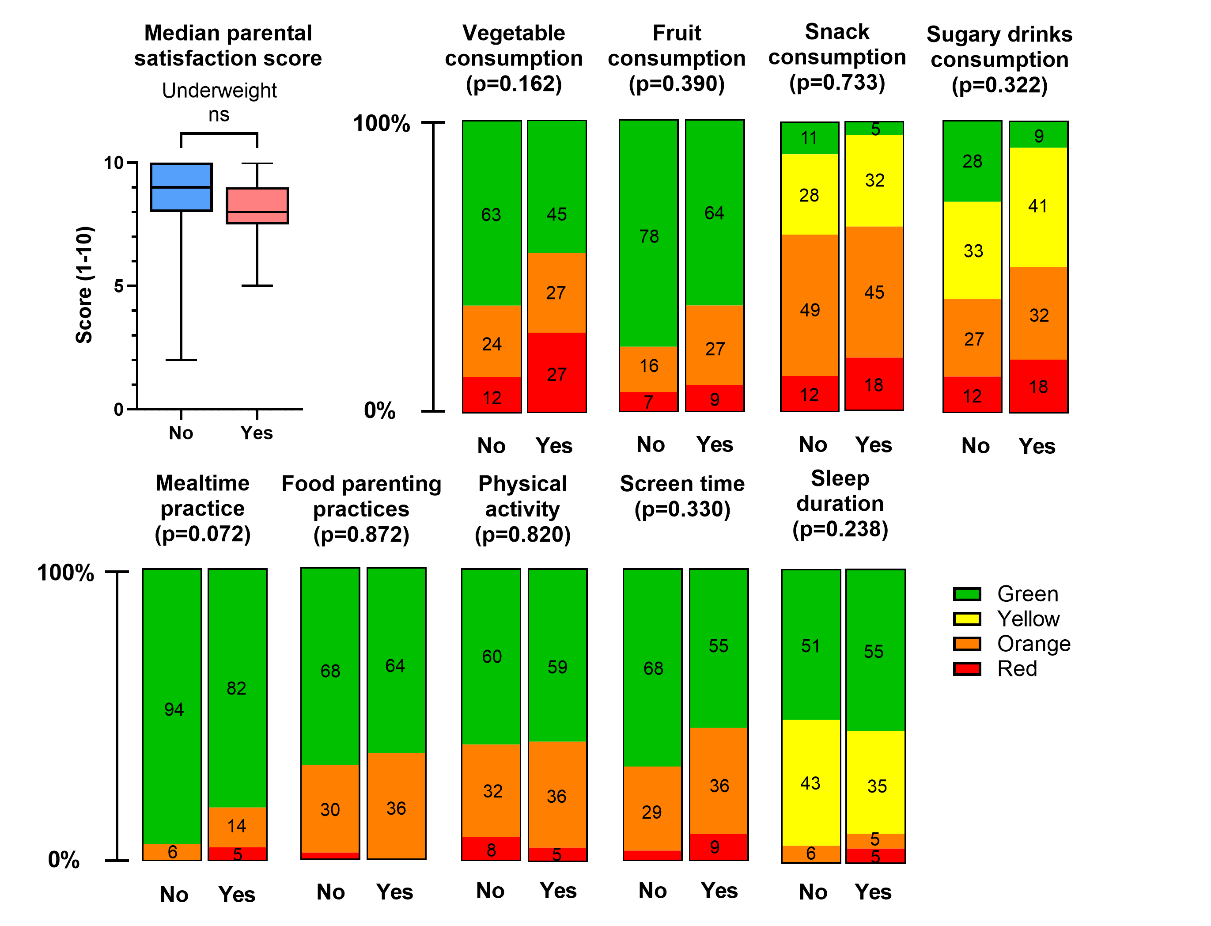


**Supplemental Fig. 3 Distribution of FLY-Kids lifestyle scores within the extremely preterm population, comparing children being underweight at 2 years’ corrected age (Yes) to children with a normal weight or overweight/obesity (No)** This figure presents the median parental satisfaction score within each group and the proportion of children in each scoring category (green, yellow, orange, red) across the nine lifestyle items. Colour-coding reflects adherence to age-specific lifestyle recommendations, with green indicating adherence and yellow, orange, and red indicating progressively lower adherence. Parental satisfaction was similar between groups and there were no significant differences in the distribution across the nine lifestyle items.


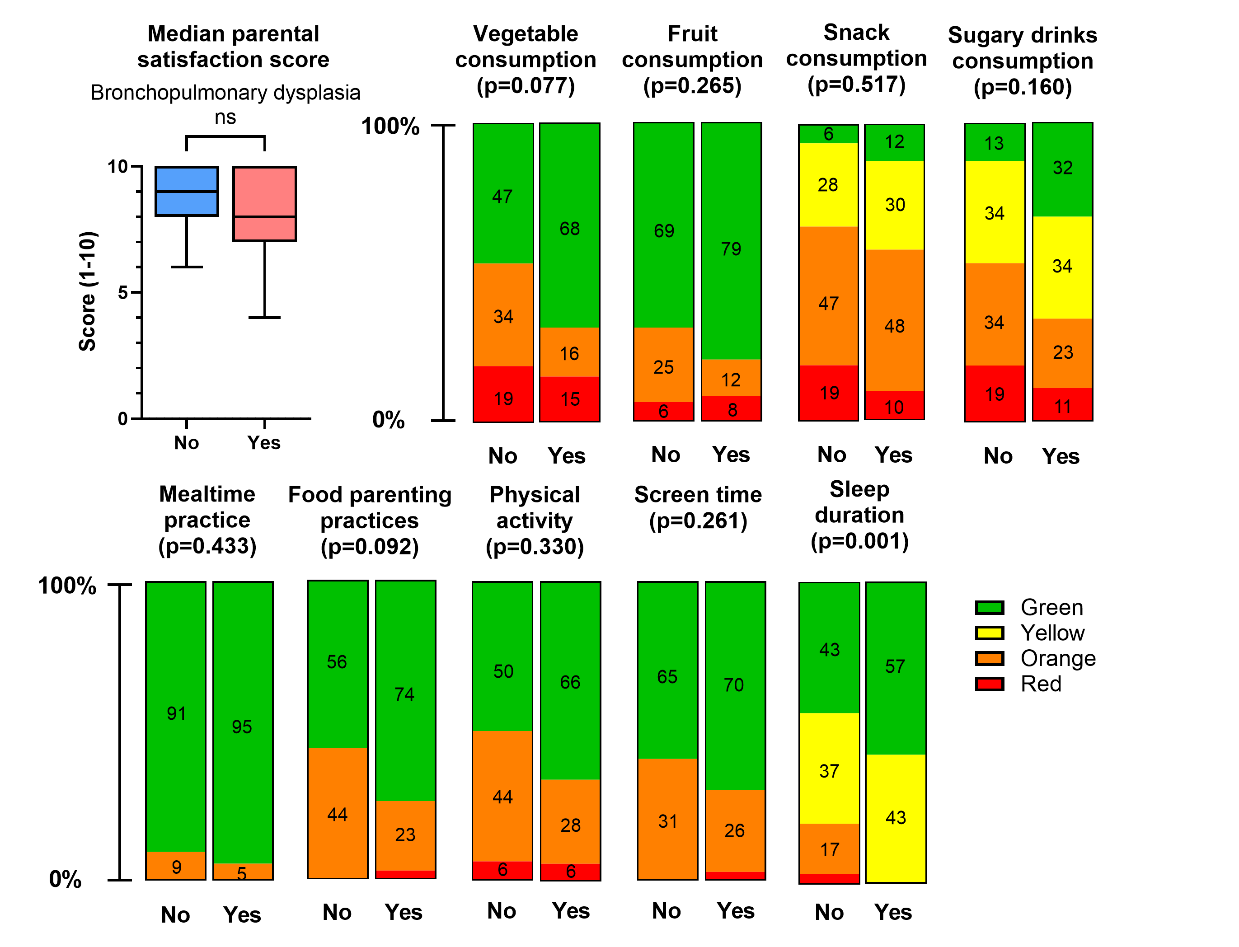


**Supplemental Fig. 4 Distribution of FLY-Kids lifestyle scores within the extremely preterm population comparing children with bronchopulmonary dysplasia (BPD, Yes) to children without BPD (No)** This figure presents the median parental satisfaction score within each group and the proportion of children in each scoring category (green, yellow, orange, red) across the nine lifestyle items. Colour-coding reflects adherence to age-specific lifestyle recommendations, with green indicating adherence and yellow, orange, and red indicating progressively lower adherence. Parental satisfaction was similar between groups. The distribution of sleep duration differed significantly between children with and without BPD, with children without BPD more frequently classified in the orange category (9 to 11 hours of sleep per 24 hours) compared to the children with BPD (adjusted standardized residual: 3.4).

**References**

1. Krijger A, Schiphof-Godart L, Elstgeest L, van Rossum C, Verkaik-Kloosterman J, Steenbergen E, Ter Borg S, Lanting C, van Drongelen K, Engelse O, et al. Development and evaluation study of FLY-Kids: a new lifestyle screening tool for young children. Eur J Pediatr. 2023;182(10):4749–57. doi:10.1007/s00431-023-05126-6.

2. Gautam N, Dessie G, Rahman MM, Khanam R. Socioeconomic status and health behavior in children and adolescents: a systematic literature review. Front Public Health. 2023;11:1228632. doi:10.3389/fpubh.2023.1228632.

3. Schonbeck Y, Talma H, van Dommelen P, Bakker B, Buitendijk SE, HiraSing RA, van Buuren S. The world's tallest nation has stopped growing taller: the height of Dutch children from 1955 to 2009. Pediatr Res. 2013;73(3):371–7. doi:10.1038/pr.2012.189.

4. Cole TJ, Lobstein T. Extended international (IOTF) body mass index cut-offs for thinness, overweight and obesity. Pediatr Obes. 2012;7(4):284–94. doi:10.1111/j.2047-6310.2012.00064.x.

5. Chou JH, Roumiantsev S, Singh R. PediTools Electronic Growth Chart Calculators: Applications in Clinical Care, Research, and Quality Improvement. J Med Internet Res. 2020;22(1):e16204. doi:10.2196/16204.

6. Fenton TR, Elmrayed S, Alshaikh BN. Fenton Third-Generation Growth Charts of Preterm Infants Without Abnormal Fetal Growth: A Systematic Review and Meta-Analysis. Paediatr Perinat Epidemiol. 2025;39(6):543–55. doi:10.1111/ppe.70035.

7. (CBS) CBvdS. Nieuwe indeling bevolking naar herkomst: Statistische Trends. 2022.

8. Waterstaat MvIe. Atlas Leefomgeving - Check je plek. 2025.

9. (CBS) CBvdS. Sociaal-economische status; scores per wijk en buurt, regio-indeling 2024 2025 [25-3-2025]. Available from: <https://www.cbs.nl/nl-nl/cijfers/detail/86092NED>.

10. Jobe AH, Bancalari E. Bronchopulmonary dysplasia. Am J Respir Crit Care Med. 2001;163(7):1723–9. doi:10.1164/ajrccm.163.7.2011060.
